# Global Cognition and Inhibition as Predictors of Dynamic Balance in Aging Populations: A Cross-Sectional Study

**DOI:** 10.3390/jcm14134754

**Published:** 2025-07-04

**Authors:** Nahid Divandari, Marie-Louise Bird, Maryam Zoghi, Fefe Vakili, Shapour Jaberzadeh

**Affiliations:** 1 Monash Neuromodulation Research Unit, Department of Physiotherapy, School of Primary and Allied Health Care, Monash University, Melbourne, VIC 3800, Australia; shapour.jaberzadeh@monash.edu; 2 Discipline of Physiotherapy, Institute of Health and Wellbeing, Federation University Australia, Churchill, VIC 3841, Australia; marielouise.bird@federation.edu.au (M.-L.B.); m.zoghi@federation.edu.au (M.Z.); 3 School of Rural Health, Latrobe University, Bendigo, VIC 3550, Australia; fatemeh.vakili.06@gmail.com

**Keywords:** executive function, cognitive domains, aging, fall prevention, exercise interventions, Y Balance Test, Timed Up and Go test, dual-task interventions, cognitive training, dynamic balance

## Abstract

**Objectives:** To identify cognitive domains predictive of dynamic balance performance in older adults and inform targeted cognitive-motor interventions aimed at improving balance and reducing fall risk. **Methods:** This cross-sectional study used hierarchical multiple regression to analyze relationships between cognitive domains and dynamic balance among 62 community-dwelling older adults (≥65 years). Balance was assessed using the Y Balance Test (YBT) and Timed Up and Go Test (TUG), while cognitive function was measured using the Mini-Mental State Examination (global cognition), Stroop Test (inhibition), N-back Test (working memory), and Deary–Liewald Reaction Time Test (processing speed). Statistical analyses were conducted using SPSS, version 28, with significance set at *p* < 0.05. **Results:** Although all cognitive domains correlated with dynamic balance, regression analyses indicated that only global cognition and inhibition were significant predictors. Specifically, global cognition significantly predicted both TUG and YBT performance, whereas inhibition uniquely predicted YBT performance (all *p* < 0.05). **Conclusions:** Our findings suggest global cognition and inhibition are key cognitive predictors of dynamic balance in older adults. Assessing these domains could identify individuals at risk of impaired balance, facilitating the design of targeted, personalized cognitive-motor interventions. Future research should investigate cognitively enriched exercise programs, including digital therapeutics and wearable technologies, to effectively target these cognitive domains, enhance balance outcomes, and promote sustained physical activity adherence in aging populations.

## 1. Introduction

The global aging population presents significant challenges for maintaining health and wellbeing in older adults, with increased fall risk as a primary concern [1]. Age-related declines in physical activity are associated with cognitive and postural decline, leading to greater frailty, increased dependency, and lower quality of life [2]. Understanding how cognitive factors influence postural control is critical for early detection of fall risk and effective intervention development.

Cognitive and dynamic balance impairments are well-established contributors to falls in older adults [3,4]. Dynamic balance relies on the integration of sensory input, reflexes, motor control, and cognitive processes to maintain postural stability and independence [5,6]. Even mild cognitive decline can adversely affect balance performance [7].

Previous research has demonstrated links between postural stability and cognitive domains such as global cognition, executive function, and processing speed in older adults [8,9,10]. These cognitive domains, especially executive function (including inhibition and working memory), are particularly vulnerable to age-related decline [11]. Brain regions such as the dorsolateral prefrontal cortex and anterior cingulate cortex, involved in cognitive regulation and postural control, also deteriorate with age [12,13], which further supports the hypothesis of shared neural networks between cognitive and motor systems [12]. This overlap may reduce the brain’s capacity to integrate motor and cognitive demands, leading to impaired balance.

Contemporary neuroscience theories offer insight into how cognitive, and motor systems interact, reinforcing the importance of cognitive integrity for balance. The neural overlap hypothesis posits that shared cortical networks between cognition and motor control, especially in the prefrontal cortex, are susceptible to age-related changes, leading to interference when executive capacity is diminished [12,14]. Additionally, the neural entrainment theory proposes that synchronizing brain oscillations across cognitive and motor regions improves coordination and efficiency by reducing cognitive load, which may also influence balance even in single-task contexts [15]. Together, these models provide a theoretical foundation for exploring cognition contributions to balance in aging.

Although prior studies have demonstrated links between cognition and balance [8,16], there remains a need to identify the most predictive cognitive domains for early detection of balance deficits and for designing targeted interventions. Such insights can support the enabling of early detection of balance impairment fall-prevention strategies, including those leveraging emerging technologies such as wearable devices, sensors, and digital therapeutics.

This study builds upon prior research by examining cognitive-motor interactions in dynamic balance. It compares performance on the Timed Up and Go (TUG) test, a widely used cognitive-motor measure, and the Y Balance Test (YBT), a complex multidirectional task that has only recently been adapted for older adults [16,17]. By exploring which cognitive domains are predictive of YBT performance, this study aims to evaluate the test’s potential relevance for assessing cognitive-motor integration and informing future fall-risk assessment strategies.

This study aims to (1) examine associations between specific cognitive domains, global cognition, inhibition, processing speed, and working memory, and dynamic balance performance in healthy older adults; (2) determine which cognitive domains significantly predict balance outcomes, beyond confounders that show a significant correlation with cognition and dynamic balance in our sample; and (3) compare cognitive predictors of performance on the Timed Up and Go (TUG) and Y Balance Test (YBT), potentially supporting its future clinical application in monitoring cognitive–motor integration and fall risk.

We hypothesize that: (1) specific cognitive domains of global cognition, inhibition, processing speed, and working memory will be significantly associated with dynamic balance performance in healthy older adults (2) these cognitive domains, particularly inhibition will significantly predict dynamic balance performance (3) cognitive domains will show stronger predictive associations with performance on the Y Balance Test (YBT) than on the Timed Up and Go (TUG) test, reflecting the YBT’s greater demands on cognitive–motor integration.

## 2. Methods

### 2.1. Participants

This observational cross-sectional study involved 62 community-dwelling older adults aged 60 and over who were functionally independent and able to walk without aid. Participants were recruited via public advertisements at universities and community centers. Of the 76 screened, 6 declined participation and 8 were excluded based on eligibility criteria. Exclusion criteria included neurological, psychological, musculoskeletal, or cardiorespiratory conditions, and any pain affecting postural control or mobility, to reduce potential confounding factors, such as cardiorespiratory conditions [18]. Eligibility was determined through a self-reported health history and a structured screening questionnaire administered by a trained physiotherapist.

### 2.2. Measurements

Demographic data were collected on age, gender, education, and falls history (Table 1). For falls history, the participants were asked in an initial survey about any instances of falling within the previous year. BMI was measured using bioelectrical impedance (Seca 804 Flat Scale with Chromed Electrodes; Seca GmbH & Co. KG, Hamburg, Germany).

Participants started by choosing a number from 1 to 4 to establish the order of cognitive tests, each linked to a specific assessment. They then proceeded to the balance tests, which were administered in a randomized sequence.

### 2.3. Cognitive Tests

Global cognition was screened using the Mini-Mental State Examination (MMSE). MMSE which includes 30 questions, evaluates orientation, attention, memory (both immediate and short-term), language abilities, and the capacity to follow verbal and written instructions. Scores range from 0 (poor) to 30 (optimal) [19].

Domain-specific cognition tests were evaluated using Psytoolkit. This platform is an effective tool for carrying out both general and psycholinguistic experiments involving complex reaction time tasks [20]. Tests were conducted as follows.

#### 2.3.1. Deary–Liewald Reaction Time Task (DLRT)

**Figure 1:**
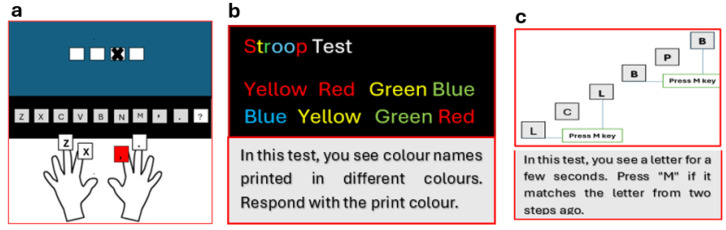
(**a**) Deary–Liewald test, (**b**) Stroop Test, (**c**) N-back test.

DLRT gauged reaction time with four white squares on a computer screen, each tied to a specific key response (“z”, “x”, “comma”, and “full stop”) [21]. Participants swiftly pressed the corresponding key when a cross appeared in a square, triggering the cross’s disappearance and the onset of another (Figure 1a). Median values (ms) from tests were used for data analysis [21]. The Deary–Liewald reaction time task is a valid and reliable measurement of processing speed and has high test–retest reliability and strong correlations with established reaction time tasks [22].

#### 2.3.2. Stroop Color–Word Test

This test assesses the capacity to inhibit cognitive interference. Participants quickly name the ink color of words on a computer screen. This test has high test–retest reliability and good internal consistency [23]. The test includes congruent (matching) and incongruent (mismatching) conditions (Figure 1b). Reaction time to incongruent conditions is analyzed as a measure of interference effects [24].

#### 2.3.3. N-Back Test

This test evaluates the working memory function [25]. Participants are shown a series of letters and must decide if the current letter matches one presented three trials earlier (Figure 1c). Their response accuracy is subsequently evaluated (Figure 1c). Previous research has reported moderate test–retest reliability for accuracy scores [26]. N-2 back task was used to assess working memory, as previous research suggests that more difficult working memory tasks show higher test–retest reliability [27].

#### 2.3.4. Balance Tests

**Figure 2:**
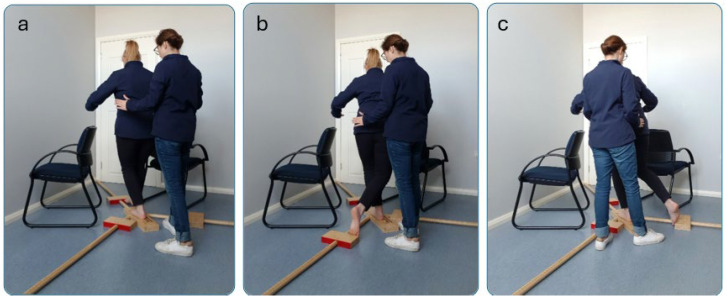
Y Balance Test. (**a**) Anterior, (**b**) posteromedial, (**c**) posterolateral, considering safety for older adults without touching the participants.

The Y Balance Test (YBT), a reliable and valid dynamic balance tool [28], was used to assess dynamic balance among older adults [29]. Participants stood barefoot on a central wooden footplate and pushed a block in three directions using their dominant leg while balancing on their non-dominant leg to increase the challenge. They performed six practice trials per direction before completing three test trials, with the mean of the test trials analyzed for each direction [29]. Adequate rest period was provided between practice and recorded trials, but not between individual trials [29]. Reach distance, measured to the nearest 0.5 cm, indicated where participants pushed the indicator block closest to the central footplate. For safety, the examiner stood one step back behind the participant. There were also two solid chairs on both sides of the YBT (Figure 2). A trial was considered invalid if the participant failed to return to the starting position, used the reaching foot to kick the plate for additional distance, and stepped on the reach indicator for support [29]. Subjects could move their arms to maintain balance. If a participant failed to maintain a unilateral stance on the platform, touched the floor, or touched the chairs and/or examiner with hands, the score was considered zero. The normalized value was determined by dividing the total of the three reach directions by three times the limb length and multiplying the result by 100. After that, the composite limb scores (average of all three directions) were averaged to generate an overall YBT-LQ composite score [29]. The measurements of the participants’ dominant lower limb length were taken in centimeters while they were lying in the supine position. The measurement involved assessing the distance from the anterosuperior iliac spine to the center of the ipsilateral medial malleolus [29].

The Timed Up and Go Test is a dynamic balance assessment [30]. Participants sat on a 45 cm chair with arms comfortably placed on their lap. The test involved timing participants as they executed a sequence of movements: standing up, walking a 3 m distance, turning around, walking back, and sitting down [10].

### 2.4. Data Management and Analysis

First, a descriptive analysis of all participants’ demographic data was performed. Then, the distribution of all data was checked for normality (*p* < 0.05). Pearson’s correlation coefficients between all demographic, cognitive, and balance variables were computed to evaluate the interrelationships between them (Table 2). Age and education were selected as potential confounders for inclusion in the regression models because they were the only confounders that exhibited significant correlations with cognitive and balance variables among all other variables such as gender, fall history, BMI, and fat mass. To investigate whether cognition contributes to a significant portion of the variance in balance beyond age and education, hierarchical multiple regression analysis (MRA) was conducted (*p* < 0.05, 0.01, and 0.001) [31]. There are 4 models as this process was repeated for each cognitive domain (Models 1 to 4) in relation to each balance test. In step one, age and education were examined as predictors. In step two, each cognitive domain was added individually to the regression equation to determine if it significantly increased the variance explained by the model (significant change in R^2^). This process was repeated for each cognitive domain in relation to each balance test [31].

Before interpreting the MRA results, several assumptions were assessed. Stem-and-leaf plots and boxplots were used to verify the normal distribution and absence of univariate outliers in each regression variable. Normal probability plots of standardized residuals and scatterplots of standardized residuals against standardized predicted values were analyzed to ensure the assumptions of normality, linearity, and homoscedasticity of residuals were met [31]. A priori power analysis was performed using G*Power version 3.1.9.7 to estimate the required sample size. Based on a medium effect size (f^2^ = 0.15), 80% power, and an alpha level of 0.05, the analysis indicated a minimum of 54 participants.

## 3. Results

### 3.1. Participants

The sample had a mean age of 74.8 years (ranging from 60 to 90) and a mean BMI of 28 (ranging from 17.3 to 42). It included 66% females and 53% had tertiary education (Table 1). Notably, 40% of participants reported recent falls.

### 3.2. Association Between Demographic Information and Cognitive and Dynamic Balance Measures

The correlation analysis revealed significant associations between education level and all cognitive as well as dynamic balance measures in the group of participants. Age showed significant associations with all dynamic balance tests and cognitive measures, excluding working memory. Falls history displayed a significant correlation solely with the TUG test. The other demographic variables exhibited no correlations with cognitive and dynamic balance measures.

### 3.3. Association Among Cognitive and Dynamic Balance Measures

All cognitive measures, including global cognition, inhibition, working memory, and processing speed, showed significant moderate association (ranging from 0.346 to 0.607, *p* < 0.01) with normalized average reach distance in YBT. It indicates that people with better performance in all aforementioned cognitive measures showed better balance function in YBT. All cognitive measures, except working memory, showed a significant moderate association (ranging from 0.405 to 0.501, *p* < 0.01) with the TUG test. Baseline cognitive and balance test results and correlations are provided in Table 2.

### 3.4. Cognitive Domains Predicting Dynamic Balance Beyond the Effect of Age and Education

Simple associations alone are not sufficient evidence for functional relationships [32]. To establish a functional relationship and determine which cognitive domain significantly contributes to the variance in dynamic balance beyond age and education, hierarchical multiple regression analyses (MRA) were conducted.

First, MRA was performed to predict dynamic balance scores based on age and education, calculating the initial R^2^ values. Next, cognitive scores were added to the model to compute new R^2^ values. The significance of the changes in R^2^ was assessed to determine whether cognitive scores accounted for additional variance in dynamic balance test scores.

In the first step of the hierarchical MRA, age, and education accounted for a significant variance in compliance. In the second step, global cognition and inhibition contributed significant additional variance, whereas processing speed and working memory did not (Table 3).

### 3.5. Cognitive Domains Predicting TUG Versus YBT

In this study, hierarchical MRA was conducted with four models, each including age and education in step one and adding a cognitive domain in step two in each model, to predict dynamic balance test scores. The regression outcomes for each cognitive domain are summarized below: Regression analyses revealed that global cognition and inhibition significantly predicted dynamic balance performance, while working memory and processing speed did not. Each regression model included age and education in step one and one cognitive domain in step two. The regression outcomes for each cognitive domain are summarized below for both TUG and YBT.

#### 3.5.1. Model 1: Inhibition (Stroop Test)

TUG: Step 1 (age and education) significantly predicted TUG scores (R^2^ = 0.544, *p* < 0.001). Adding inhibition in step 2 resulted in a non-significant change in R^2^ (ΔR^2^ = 0.004), although the overall model remained significant (F(2,62) = 23.47, *p* = 0.0008, R^2^ = 0.548).

YBT: Step 1 significantly predicted YBT (R^2^ = 0.399, *p* < 0.001). Adding inhibition in step 2 led to a significant change in R^2^ (ΔR^2^ = 0.050, *p* = 0.02), indicating that inhibition significantly predicted YBT beyond age and education.

#### 3.5.2. Model 2: Global Cognition (MMSE)

TUG: Step 1 was significant. Adding global cognition significantly improved the model (ΔR^2^ = 0.051, *p* = 0.000), with the final model explaining 59.5% of the variance (F(2,62) = 24.22, *p* = 0.0006, R^2^ = 0.595).

YBT: Step 1 was significant. Adding global cognition resulted in a significant ΔR^2^ = 0.068 (*p* = 0.009), with a final R^2^ = 0.467. Thus, global cognition significantly predicted both TUG and YBT.

#### 3.5.3. Model 3: Working Memory (N-back)

TUG: Step 1 was significant. Adding working memory in step 2 did not change the model (ΔR^2^ = 0.000, *p* > 0.05).

YBT: Step 1 was significant. Step 2 led to a small, non-significant change (ΔR^2^ = 0.031, *p* > 0.05), indicating no predictive value.

#### 3.5.4. Model 4: Processing Speed (Deary–Liewald Reaction Time Test)

TUG: The final model remained significant (F(2,62) = 24.22, *p* = 0.0008), but the addition of processing speed did not improve the model (ΔR^2^ = 0.004).

YBT: The model remained significant overall (F(2,62) = 17.96, *p* = 0.0009), but adding processing speed resulted in a negligible and non-significant change in R^2^ (ΔR^2^ = 0.001).

These findings address Aim 2 and 3 of the study by identifying global cognition and inhibition as the cognitive domains that significantly predicted dynamic balance performance beyond age and education and comparing this between TUG and YBT.

The unique contributions of inhibition and global cognition to dynamic balance were further supported by effect size estimates. Inhibition showed a small-to-moderate effect (f^2^ = 0.091), while global cognition demonstrated a slightly larger small-to-moderate effect (f^2^ = 0.128), indicating practical significance despite modest R^2^ changes. Cohen (1988) defined small, medium, and large effect sizes for multiple regression as f^2^ = 0.02, 0.15, and 0.35, respectively [33].

## 4. Discussion

This study addressed three key aims. Firstly, it identified significant associations between cognitive domains and dynamic balance performance. Secondly, it demonstrated that global cognition and inhibition significantly predict dynamic balance beyond age and education, highlighting their unique contributions. Finally, it compared cognitive predictors of YBT and TUG performance, showing that YBT was significantly predicted by global cognition and inhibition, whereas TUG performance was predominantly influenced by global cognition. These findings suggest that global cognition and inhibition may be relevant factors to consider in the assessment of balance in older adults, though further validation is needed before clinical application. Incorporating tasks that specifically challenge these cognitive domains, such as dual-task exercises emphasizing attentional control and cognitive-motor integration, may enhance the effectiveness of dynamic balance interventions. Additionally, these targeted exercises could be effectively delivered and monitored using digital therapeutics and wearable technologies, aligning closely with personalized, technology-enhanced exercise programs for aging populations.

Regarding aim one, global cognition, inhibition, and processing speed exhibited significant moderate associations with both dynamic balance tests, aligning with previous research [34]. Interestingly, the N-back test, assessing working memory, showed a significant association only with YBT, differing from previous findings [35]. Given our robust study population, the TUG test may not have been sufficiently challenging cognitively, possibly explaining why working memory did not significantly associate with TUG performance.

Regarding aim two, hierarchical regression analysis demonstrated that cognitive function, age, and education collectively predict dynamic balance significantly. Notably, adding global cognition and inhibition significantly improved prediction (significant increase in R^2^ values), highlighting their unique contributions—global cognition for both YBT and TUG and inhibition specifically for YBT. These abilities are essential during dynamic balance tasks, which demand real-time adjustment and coordination.

To our knowledge, this study highlights global cognition and inhibition as statistically significant predictors of dynamic balance in older adults, whereas prior research primarily explored balance-predicting cognition or general associations [36,37]. The observed inconsistencies between our findings and those reported by Zhao et al. (2022) and Won et al. (2014) may be attributable to methodological differences such as participant selection and assessment procedures, underscoring the importance of employing standardized protocols in future research [10,37]. Zhao et al. (2022), for instance, enrolled participants from community health service centers, in contrast to our study, which included a robust, community-dwelling cohort [10]. Additionally, Kwan et al. (2011) utilized the Timed Up and Go (TUG) test, instructing participants to walk at their own comfortable pace, whereas our study instructed participants to walk as fast and safely as possible [38]. These methodological differences underscore the need to consider multiple factors influencing study outcomes.

Age-related cognitive decline may partially explain why global cognition and inhibition predict dynamic balance. Previous longitudinal research suggests cognitive deterioration often precedes mobility limitations, potentially reducing physical activity and subsequent balance ability [39,40]. Further longitudinal studies are needed to confirm causality and deepen the understanding of cognitive-mobility relationships.

Previous studies have associated better balance with superior Stroop Test performance, though typically focusing on balance as a predictor of cognitive function, though these studies focused on balance predicting cognition [41,42]. Executive function, particularly inhibition, is crucial for coordinating movement and interacting with the environment, suggesting it plays a significant role in maintaining balance through sensory integration processes [43,44]. Structural changes in brain regions linked to inhibition, such as the middle frontal gyrus and basal ganglia, may underpin age-related postural control declines [45]. Therefore, integrating inhibition assessments such as the Stroop Test in balance evaluations could offer valuable insights for identifying older adults at heightened risk of balance impairment.

However, our regression analysis revealed inhibition did not independently predict TUG performance. Given the robustness of our sample, the TUG may not have presented sufficient challenge to reveal significant associations with inhibition. Consistent with Berryman et al. (2013), who found a correlation between cognitive flexibility but not inhibition (Stroop) and TUG performance, our findings suggest the TUG may not fully capture cognitive-motor integration in robust, healthy older adults [41]. This highlights the importance of task selection tailored to cognitive challenge in older populations [46].

The predictive association between global cognition and dynamic balance performance may be explained by the underlying neuroanatomical connectivity shared by cognitive and motor systems. Neuroimaging studies have demonstrated that MMSE performance correlates with structural integrity in key brain regions such as the hippocampus, parahippocampal gyrus, cingulate cortex, and middle temporal gyrus [47]. Among these, the cingulate cortex, particularly its anterior, midcingulate, and posterior subdivisions, acts as a central integrative hub for linking reward processing, memory, spatial orientation, and motor planning [48]. This broader network underlies both the cognitive functions assessed by the MMSE, such as memory, attention, and orientation, and motor functions related to postural control. Therefore, the MMSE may serve as a proxy indicator of the structural and functional integrity of a distributed neurocognitive-motor system, helping to explain its predictive value for dynamic balance in older adults.

Inhibitory control, the ability to suppress automatic, inappropriate, or undesired responses, is essential for goal-directed behavior and everyday functioning [49]. This relies on several brain regions, particularly the prefrontal cortex and anterior cingulate cortex, which play a central role in its regulation [50]. Research over the past two decades has consistently shown that the right lateral prefrontal cortex is especially important in supporting general inhibitory control [51]. Functional MRI studies in healthy individuals [52,53] have demonstrated increased activity in the right inferior frontal gyrus (rIFG) and anterior insula (aIns), reinforcing their role as core neural substrates for inhibition. Aging has been associated with decreased activation in these regions, along with compensatory increases in other areas [13,54]. This decline in network efficiency may help explain the predictive role of inhibition in balance performance observed in this study. Effective inhibition supports postural control by enabling individuals to suppress competing or maladaptive motor responses, especially during dynamic tasks that require quick adaptation. Anatomical studies further support this relationship. Lu et al. (1994) demonstrated that the dorsolateral prefrontal cortex—a key region involved in inhibitory control—is interconnected with several premotor areas, including the ventral premotor cortex, supplementary motor area, and cingulate motor areas, in a topographically organized manner [55]. These connections suggest that cognitive processes regulated by the prefrontal cortex, such as inhibition, can directly influence motor planning and execution. Complementing this, Fine and Hayden (2021) proposed that the prefrontal cortex itself functions as a premotor structure, organized hierarchically to optimize action selection [56]. This theoretical perspective aligns with the idea that inhibitory capacity supports the precision and coordination of movement, especially during complex postural tasks requiring suppression of automatic or maladaptive responses. As such, strong inhibitory capacity is likely to contribute to better movement coordination and balance stability in older adults.

Regarding aim three, this study compared how cognitive domains predict performance on the YBT versus TUG. Results indicated that YBT performance was significantly predicted by both global cognition and inhibition, highlighting its sensitivity to tasks requiring complex cognitive-motor integration. In contrast, TUG performance was predominantly influenced by global cognition and was significantly associated with a history of falls, reinforcing its utility in assessing functional mobility and real-world fall risk. These findings underscore the clinical importance of incorporating cognitive evaluations into balance assessments to identify older adults at risk of dynamic balance impairments. Clinically, while the TUG remains valuable for evaluating functional mobility and practical fall risk, the YBT provides complementary insights, particularly among populations with higher baseline mobility.

It should be mentioned that although the ΔR^2^ values in this study were modest, prior research shows that even subtle deficits in inhibition can affect balance in older adults [57,58]. Also, frail older adults demonstrated significantly poorer performance in both simple gait and dual motor–“tasks” [59]. Therefore, the predictive value of cognitive measures is likely to be even greater in more vulnerable populations, highlighting the value of cognitive assessments in fall risk screening.

## 5. Clinical Implications

The findings from this study highlight the predictive role of specific cognitive domains, inhibition and global cognition, in dynamic balance performance. These findings may inform future research on fall prevention strategies in both community and clinical contexts. In clinical settings, incorporating brief cognitive screening tools (e.g., Stroop Test, MMSE) into routine balance assessments may help physiotherapists and other health professionals identify older adults at increased risk of balance impairment, even before overt motor deficits are evident. This is especially relevant in time-limited environments such as outpatient clinics or aged care facilities.

In community settings, these findings support the design of dual-domain interventions that simultaneously target cognitive and motor functions. This approach may enhance motor learning and improve balance under real-life conditions where attentional demands are present.

Furthermore, the evidence that global cognition and inhibition relate to balance performance even in healthy, high-functioning older adults underscores the importance of early prevention. Intervening before cognitive or motor decline becomes clinically apparent could delay or reduce fall risk and associated morbidity.

Additionally, it is crucial to select tasks that are appropriately challenging for each individual. Therefore, an outcome measure to assess the challenge level of cognitive and balance tasks before tailoring them to clients is necessary. Tailoring interventions based on cognitive profiles may optimize balance rehabilitation, particularly for individuals at risk of mobility decline and falls.

As this study was cross-sectional, future research should focus on longitudinal designs to establish causality and explore how incorporating these cognitive domains into dual-task balance training can impact balance outcomes. Investigating the effects of such targeted interventions will help determine the most effective strategies for promoting long-term adherence to physical activity in older adults. Also, it is recommended that future studies include participants with varying levels of frailty to better understand how frailty status may modify associations between determinants and health outcomes.

## 6. Limitations

This study has some limitations that should be considered when interpreting the findings. First, the cross-sectional design does not allow for the establishment of causal relationships between cognitive domains and dynamic balance. Although the regression models identified significant predictors, the directionality of these relationships needs to be explored in longitudinal studies.

Second, the performance on the TUG test may not have been sufficiently challenging for this relatively high-functioning group, potentially reducing its sensitivity to detect associations with specific cognitive domains. While YBT proved to be a safe and effective tool for assessing dynamic balance and cognitive–motor integration in this group, future studies should consider more complex alternatives, such as instrumented TUG.

Third, while the study incorporated multiple cognitive tests, including the MMSE, Stroop Test, and N-back test, it did not account for other potentially influential factors, such as sensory deficits, muscle strength, sex, physical activity level, or unreported medical conditions, which could also affect dynamic balance. Another limitation of this study is the omission of physical health factors such as obesity, which are known to impact both cognitive functioning and balance performance [60,61]. Future studies should consider incorporating these variables or conducting sensitivity analyses to examine their potential influence. Our participants were generally healthy and functionally independent, which may limit generalizability to frailer populations. However, the predictive value of the cognition–balance relationship may be stronger in frail older adults, as frailty appears to amplify cognitive–motor interactions. For instance, frail older adults demonstrated significantly poorer performance in both simple gait and dual motor–cognitive tasks [59]. Future research should explore whether similar patterns exist in more vulnerable cohorts to inform targeted fall prevention strategies.

## 7. Conclusions

This study identified significant associations between cognitive domains and dynamic balance performance in robust older adults. Global cognition and inhibition emerged as statistically significant predictors of balance, beyond the effects of age and education. The Y Balance Test (YBT) was sensitive to both global cognition and inhibition, whereas the Timed Up and Go (TUG) primarily reflected global cognitive function. Clinically, these findings may support the use of brief cognitive assessments, such as the MMSE and Stroop Test, to help identify older adults at risk of impaired balance. The YBT, in particular, may offer greater potential as a clinical tool for monitoring cognitive–motor integration and fall risk in this population. However, these implications should be interpreted with caution, as they require the evaluation of future studies. Future longitudinal studies with larger and more diverse samples are needed to confirm these findings and explore their relevance in clinical settings.

## Figures and Tables

**Table 1:** Characteristics of participants.

Variables	Number (%)	Mean ± SD
Age	62	74 ± 8.6
BMI	62	28.1 ± 5.7
Education		
High school	33 (53.2%)	
College/Diploma	11 (17.7%)	
Bachelor	10 (16.1%)	
Master/PhD	8 (12.9%)	
Sex		
Female	41 (66%)	
Male	21 (34)	
Falls history		
Yes	25 (40%)	
No	37 (60%)	

**Table 2:** Descriptive data on cognitive and dynamic balance measures (N = 62), and the associations observed between cognitive domains and dynamic balance.

Variables	Mean	SD	1	2	3	4	5	6
1—YBT	56.9	17.3	1					
2—TUG	6.7	1.5	−0.607 **	1				
3—MMSE	28.2	1.6	0.473 **	−0.478 **	1			
4—Deary–Liewald	672.4	141.7	−0.379 **	0.501 **	−0.315	1		
5—Stroop	1297.5	191.6	−0.567 **	0.405 **	−0.345 **	0.527 **	1	
6—N-back	59.5	17.5	0.346 **	−0.221	0.237	−0.144	−0.295 *	1

* Correlation is significant at the 0.05 level, ** Correlation is significant at the 0.01 level, (N = 62); N: Number of participants; Stroop: Stroop color word; MMSE; Mini-Mental State Examination; TUG: Timed Up and Go; YBT: Y Balance Test.

**Table 3:** Hierarchical regression results for cognitive domains predicting dynamic balance.

Model 1			Model 2		
Independent Variables	YBT	TUG	Independent Variables	YBT	TUG
Step 1: Background variables			Step 1: Background variables		
Age	−0.558 ***	0.627 ***	Age	−0.558 ***	0.627 ***
Education	0.234 *	−0.318 ***	Education	0.234 **	−0.318 ***
Step 2: Cognitive variable			Step 2: Cognitive variable		
Age	−0.398 **	0.673 ***	Age	−0.463 **	0.544 ***
Education	0.176	−0.335 ***	Education	0.206 *	−0.293 ***
Inhibition	0.284 *	−0.082	Global cognition	0.280 **	−0.243 **
R^2^ step 1 (Age + Edu)	0.399	0.544	R^2^ step 1 (Age + Edu)	0.399	0.544
R^2^ step 2 (Age + Edu + Inhibition)	0.449	0.548	R^2^ step 2 (Age + Edu + global cognition)	0.467	0.595
R^2^ change	0.050*	0.004	R^2^ change	0.068 **	0.051 **
**Model 3**			**Model 4**		
**Independent Variables**	**YBT**	**TUG**	**Independent Variables**	**YBT**	**TUG**
Step 1: Background variables			Step 1: Background variables		
Age	−0.525 ***	0.627 ***	Age	−0.558 ***	0.627 ***
Education	0.234 *	−0.318 ***	Education	0.234 *	−0.318 ***
Step 2: Cognitive variable			Step 2: Cognitive variable		
Age	−0.525 ***	0.626 ***	Age	−0.539 ***	0.561 ***
Education	0.189	−0.317 ***	Education	0.227	−0.292 **
Working memory	0.185	−0.004	Processing speed	−0.039	0.132
R^2^ step 1 (Age + Edu)	0.399	0.544	R^2^ step 1 (Age + Edu)	0.399	0.529
R^2^ step 2 (Age + Edu + Working Memory)	0.43	0.544	R^2^ step 2 (Age + Edu + Processing speed)	0.4	0.533
R^2^ change	0.031	0	R^2^ change	0.001	0.004

Age, education level, and cognitive domain are predictors. YBT: Y Balance Test; TUG: Time Up and Go, Edu: Education; β: standardized regression coefficient; CI: confidence interval; R^2^: The coefficient of determination; ΔR^2^: change in R^2^ (improvement in R-square when the second predictor is added) * *p* < 0.05, ** *p* < 0.01, and *** *p* < 0.001.

## Data Availability

The data supporting the findings of this study are available from the corresponding author upon reasonable request.
